# The Anatomy Aud Development of the Rodent Ulcer

**Published:** 1872-12

**Authors:** 


*The Anatomy and Devolvopment of the Rodent Ulcer.* A Boylston Medical Prize Essay for 1872. By J. COLLINS WARREN, M. D. Boston : Little, Brown & Co., 1872.

The term rodent ulcer will suggest to many minds a question as to the meaning of the author it being something that is but little understood, or, at least, little treated of by American or European writers. That it has been in many instances treated of as simply common epithclial cancer, and under this head passed over or but lightly touched, is undoubtedly the case. Some pathologists have noticed its peculiar character and spoken of it to some extent, but their efforts have only tended in many instances to produce confusion. No less than eight different names are mentioned by the author as being applied to it by different writers. Five cases are reported, and the microscopical appearances given by the author. The cases are well reported, and the microscopical appearances exceeding well shown in two plates, which close the book. The profession are under obligations to Dr. Warren for his careful and minute research and well-recorded observations concerning the pathology and development of this disease.

